# Characteristics and surgical outcomes of combat blast-related full-thickness traumatic macular holes: insights from war eye trauma in Ukraine

**DOI:** 10.1186/s40942-026-00816-3

**Published:** 2026-03-28

**Authors:** Andrii Ruban, Vitalyi Prudyus, Anna Zolnikova, Beáta Éva Petrovski, Goran Petrovski, Susanne Binder, Andrzej Grzybowski, Lyubomyr M. Lytvynchuk

**Affiliations:** 1 Center of Clinical Ophthalmology, Kyiv, Ukraine; 2 Department of Ophthalmology, Oslo University Hospital, Oslo, Norway; 3 Institute of Endocrinology, Lithuanian University of Health Sciences, Kaunas, Lithuania; 4 Center for Eye Research and Innovative Diagnostics, Department of Ophthalmology, Institute of Clinical Medicine, Faculty of Medicine, University of Oslo, Oslo, Norway; 5 Department of Ophthalmology, University of Split School of Medicine, University Hospital Centre, Split, Croatia; 6 UKLONetwork, University St. Kliment Ohridski-Bitola, Bitola, North Macedonia; 7 Department of Ophthalmology, Sigmund Freud University of Vienna, Vienna, Austria; 8 Department of Ophthalmology, University of Warmia and Mazury, Olsztyn, Poland; 9 Institute for Research in Ophthalmology, Foundation for Ophthalmology Development, Poznan, Poland; 10 Department of Ophthalmology, Justus Liebig University Giessen, Eye Clinic, University Hospital Giessen and Marburg GmbH, Campus Giessen,Friedrichstrasse 18, 35392 Giessen, Germany; 11 Karl Landsteiner Institute for Retinal Research and Imaging, Vienna, Austria

**Keywords:** Blast, Combat ocular trauma, Full-thickness macular hole, Blast-related traumatic full-thickness macular holes (BRTMH), Pars plana vitrectomy, Prognostic factors

## Abstract

**Background:**

This study analyzes the clinical features and surgical outcomes of combat blast–related traumatic full-thickness macular holes (BRTMH) secondary to war-related ocular trauma in Ukraine, managed at a single civilian center.

**Methods:**

Thirteen patients (14 eyes) with diagnosis of BRTMH treated with pars plana vitrectomy (PPV) were recruited for this retrospective, consecutive case, interventional study. Surgery outcome-related factors including face-down positioning were assessed and statistically analyzed.

**Results:**

Blast trauma was the cause of injury in all (100%) of the MH cases, wearing no eye protection. Four eyes (28.6%) with MH were secondary to an open-globe, whereas ten eyes (71.4) were related to a closed-globe injury. MH closure was achieved in all cases (100%) after the primary surgery. The median (IQR) ocular trauma score (OTS) was 68 (56–75), while the time interval from injury to surgery was 41 (19 to 71) days. The median (IQR, interquartile range 25–75%) Minimum Linear Diameters of the MHs (µm) was 682 μm (532–889), while nine out of fourteen eyes (64.3%) had BRTMH > 600 μm. There was a direct correlation of postoperative visual acuity at 1 month with the OTS score (ρ = 0.51, *p* = 0.03) and preoperative visual acuity (LogMar) (ρ = 0.72, *p* = 0.002), and an inverse correlation with the size of the MH (ρ = -0.63 *p* = 0.008).

**Conclusion:**

Combination of different surgical approaches with minimization of postoperative face-down position time allows to achieve high anatomical and functional results being safe and highly acceptable for wounded patients with BRTMH.

**Supplementary Information:**

The online version contains supplementary material available at 10.1186/s40942-026-00816-3.

## Background

In modern war combat eye injuries are common among the soldiers and reach up to 10–15% of all trauma cases [1, 2]. Advances in weapon systems and battle tactics may explain the increase in the incidence and severity of eye injuries. Most eye injuries are generally secondary to blast trauma, which are characterized by extremely high kinetic energy of the striking objects (cruise missiles, bombs, first-person view drones (FPV-drones), fragments of shells, grenades and mines) [3].

Unlike civil injury, combat eye trauma is defined as a complex eye polytrauma where the eye is only one of the components of the systemic polytrauma. Such eye injuries are frequently bilateral and associated with globe perforation, retained intraocular foreign bodies (IOFBs) and visual impairments [4]. Blast-related closed globe injuries can result in a spectrum of severe macular and optic nerve injuries among which macular holes (MH) take an important role [5].

Although overall 52% of all combat ocular trauma injuries retain 20/40 or better best corrected visual acuity (BCVA) [6], patients with traumatic MH have a much poorer visual prognosis [7]. Combat eye injuries lead to a significant financial, social and psychological costs to the individual and the society, and often render combatants unfit for military service and many civil occupations [8].

This study reports the anatomical and visual outcomes as well as determines the risk factors influence on postoperative BCVA of combat blast-related traumatic macular hole surgery in those wounded during the war in Ukraine.

## Methods

### Ethical statement

This retrospective, consecutive, interventional case series study was conducted at the “Center of Clinical Ophthalmology” (Kyiv, Ukraine) from November 2022 to October 2024 and examined patients injured during full scale Russian aggression. This study adhered to the Declaration of Helsinki, and ethics approval was obtained from the local research ethics committee at “Center of Clinical Ophthalmology” (Kyiv, Ukraine) (Protocol №2022-8-21). All patients were informed about all risks and benefits of the surgical treatment and signed a written Consent to Participate declaration. The content of this article has been reviewed and approved in accordance with operational security protocols.

All patients in this study were examined and followed at the Center of Clinical Ophthalmology (Kyiv, Ukraine) until either their return to active duty or their transfer to the Veterans Affairs medical system.

### Patients and clinical examination

Fifteen patients (16 eyes, 13 soldiers and 2 civilians) with a combat blast-related traumatic macular hole (BRTMH) were enrolled to this study. Two patients (2 eyes) were excluded from the study: one patient was unable to attend follow-up examinations after surgery, and the other had surgery cancelled due to spontaneous closure of the macular hole. The final study group thus comprised 13 cases (14 eyes).

The main outcome measures included full-thickness macular hole (FTMH) closure rates, final best-corrected visual acuity (BCVA), Ocular Trauma Score (OTS), and complications related to surgical intervention.

Eye injuries were classified as closed or open-globe in accordance with the Birmingham Eye Trauma Terminology (BETT) [9]. The ocular trauma score was retrospectively calculated on the basis of six variables as proposed by F.Kuhn: initial VA, rupture, endophthalmitis, perforating injury, retinal detachment, and RAPD. The scores were stratified into five categories that gave the predictabilities of the final BCVA [10].

After the injury (similar to the North Atlantic Treaty Organization (NATO) standards), soldiers were evacuated to a first large medical center located to the rear of the battle zone (Echelon 3), where initial eye evaluation and primary surgical repair were performed by an ophthalmologist as quickly as possible. If there were no severe non-ocular injuries, majority of soldiers once stabilized after the primary ocular surgery arrived at the Echelon 4 Medical Center level care to the hospitals of territorial hospital bases (THBs) of the Ministry of Health, National Medical Clinical Center of the Ministry of Internal Affairs, regional Military Medical Clinical Centers (MMCCs) or the National Military Medical Clinical Center “Main Military Clinical Hospital”. Civilians wounded were hospitalized to the hospitals of the Ministry of Health of Ukraine.

The visual acuity was documented during the evacuation process upon patient stabilization when communication was feasible. Initial visual acuity for intubated and non-communicative soldiers was recorded once they were extubated and able to participate in the examination.

All patients had a complete ophthalmic examination at the “Center of Clinical Ophthalmology” before and after surgery, including measurement of BCVA (decimal), applanation tonometry, slit-lamp examination, fundus ophthalmoscopy, ultrasound B-scan imaging. The use of eye protection was documented as “yes” or “no” for all cases.

### Optical coherence tomography

Retinal cross-sectional images before and after surgery were obtained using swept source optical coherence tomography system (SS-OCT) (Triton, TOPCON Corporation, Tokyo, Japan) with horizontal scans through the center of the MH being analyzed. Minimal Linear Dimension (MLD) was determined using a protocol previously described by the International Vitreomacular Traction Study Group [11]. The minimum width of the MH was measured at the narrowest hole point in the mid-retina of the foveal horizontal B-scan, using the OCT caliper function, as a line drawn parallel to the retinal pigmented epithelium (RPE). MH closure was defined by SS-OCT as the complete disappearance of the hole and absence of neurosensory defect over the fovea. Flat-open and elevated-open MHs were considered as surgical failures.

### Surgical procedure

All surgeries were performed by one experienced vitreoretinal surgeon (AR). A standard 25G PPV was performed in all cases either under retrobulbar anesthesia or general anaesthesia using the Constellation^®^ Vision System (Alcon, ForthWorth, TX, USA) under a non-contact viewing system (Topcon, Tokyo, Japan). After the pars plana core vitrectomy (PPV), posterior vitreous body was detached and removed with or without triamcinolone-assisted visualization up to the vitreous base. Membrane Blue Dual (DORC, Zuidland, the Netherlands) was then injected to stain the internal limiting membrane (ILM) for approximately 30 s, followed by its removal. The ILM was peeled in at 360-degree manner 1.5–2.0 disk diameters (DD) around the hole. Air–fluid exchange was facilitated with the back-flush cannula and drainage of subretinal fluid was also performed through the MH.

Individually, depending on the MLD size and degree of subretinal adhesion, the additional surgical options included: inverted ILM flap technique (superior, temporal or multilayers) and subretinal fluid application with ccentripetal rretinal displacement.

To mobilize the retinal tissue, subretinal injections of balanced salt solution (BSS) (Alcon Laboratories, Inc, Fort Worth, TX) were performed within the major retinal vascular arcades using a subretinal PolyTip cannula 25/41-gauge (MedOne Surgical, Inc, Sarasota, FL) connected to the syringe filled with BSS. Subretinal injection was performed in a four-point manner through puncture retinotomies at a distance of three to four DD from the MH edges to create local retinal detachments. The subretinal injections were controlled to the point when all blebs were connected with the MH. Perifoveal centripetal macular displacement of the detached retinal tissue was performed as a gentle massage using a backflush cannula (DORC, VC Zuidland, the Netherland). During this procedure, the infusion pressure was temporarily reduced with vitrectomy system from 30 mmHg down to 15 mmHg. In cases with subretinal chorioretinal scarring, a dissection of retinal-choroidal adhesion was performed using 25-gauge needle (Becton; Dickinson and Company, Heidelberg, Germany) or Flex loop (Alcon).

Each surgery was completed by injection of a gas–air mixture of 20% sulfur hexafluoride (SF6) or 16% hexafluoroethane (C2F6) or silicon oil (Alchimia, Ponte San Nicolo PD, Italy).

All the patients were ordered to strictly keep face-down posturing for 24 h after surgery. SS-OCT examination was performed on postoperative day 1. When closure of the FTMH was confirmed at day 1, posturing was stopped. If not, face down position (FDP) was continued for 2 additional days.

In three cases with concomitant rhegmatogenous retinal detachment, temporary perfluorocarbon liquid (PFCL) tamponade was performed with indirect PFCL-air-silicon oil (SO) or PFCL-air-gas exchange.

The postoperative protocol consisted of routine topical antibiotic and anti-inflammatory agents (dexamethasone and non-steroidal anti-inflammatory drugs (NSAID)).

### Statistical methods

The BCVA was recorded as decimal value and converted to the logarithm of minimal angle of resolution (logMAR) for statistical analysis. The description used the median (interquartile range (IQR)) values. Quantitative indicators were checked for compliance with the normal distribution law by the Kolmogorov–Smirnov criterion. When paired comparison, the Wilcoxon test (W) was used, the relationship between indicators was checked by the one-sided Spearman test (ρ). Qualitative indicators in the groups were compared using contingency tables, presence of connection - Fisher’s exact test (F)). A p-value of less than 0.05 was considered to be statistically significant. The data were processed using STATISTICA 8 software (StatSoft, Inc, Tulsa, OK, USA).

## Results

From November 2022 to October 2024, three hundred fifty-four patients (365 eyes) with combat globe injuries turned to the “Center of Clinical Ophthalmology”, Kyiv, Ukraine for quaternary care. Sixteen of the 365 eyes (4.4%) were diagnosed with a full-thickness MH.

Overall, thirteen patients (14 eyes) with a combat-related traumatic MH were included in the study. The median age was 32 years (range: 25.5–43.0 years) and a median (IQR) follow-up was 75 (43–175) days. Males accounted for 100% of BRTMH and one of them (7.6%) had bilateral MHs. The median (IQR) of the MLD of the MHs was 682 μm (532–889) and nine out of fourteen eyes (64.3%) had MH > 600 μm.

**Fig. 1:**
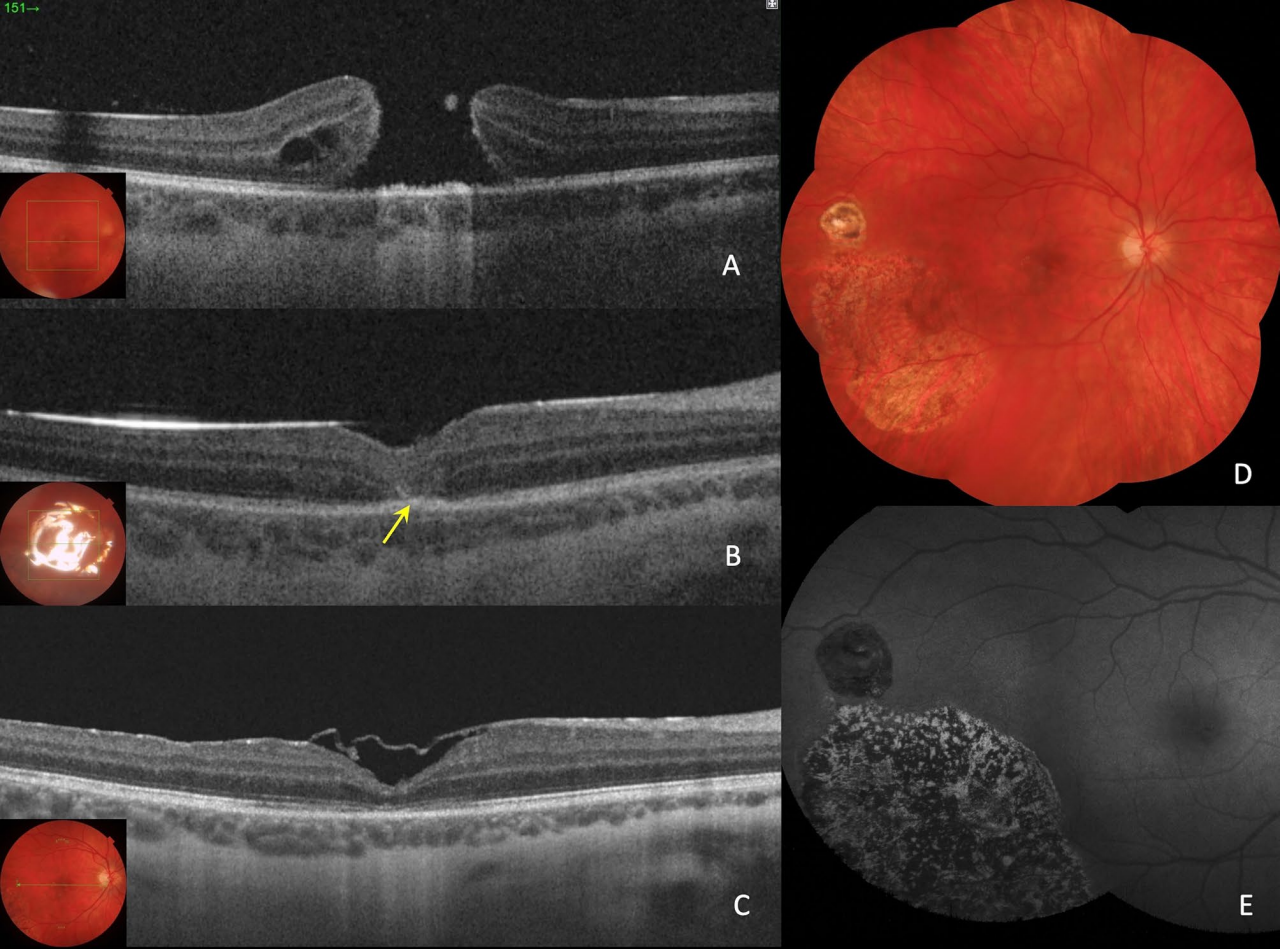
Case 1: 23-year-old man with BRTMH was treated with phacovitrectomy 25G and inferior ILM peeling + superior ILM inverted flap + subretinal fluid application + centripetal retinal displacement + C_2_F_6_ gas. (**A**). His preoperative MLD was 768 μm, and the BCVA was 0.08 decimal (**B**). One day after surgery, the MH was closed, but the ELM line and the EZ line were noticeably interrupted (yellow arrow) (**C**). One month after surgery, the BCVA was 0.4 decimal, the ELM line completely restored but the EZ line were partially restored. (**D**) Postoperative fundus image. (**E**) Postoperative fundus autofuorescence image

**Fig. 2:**
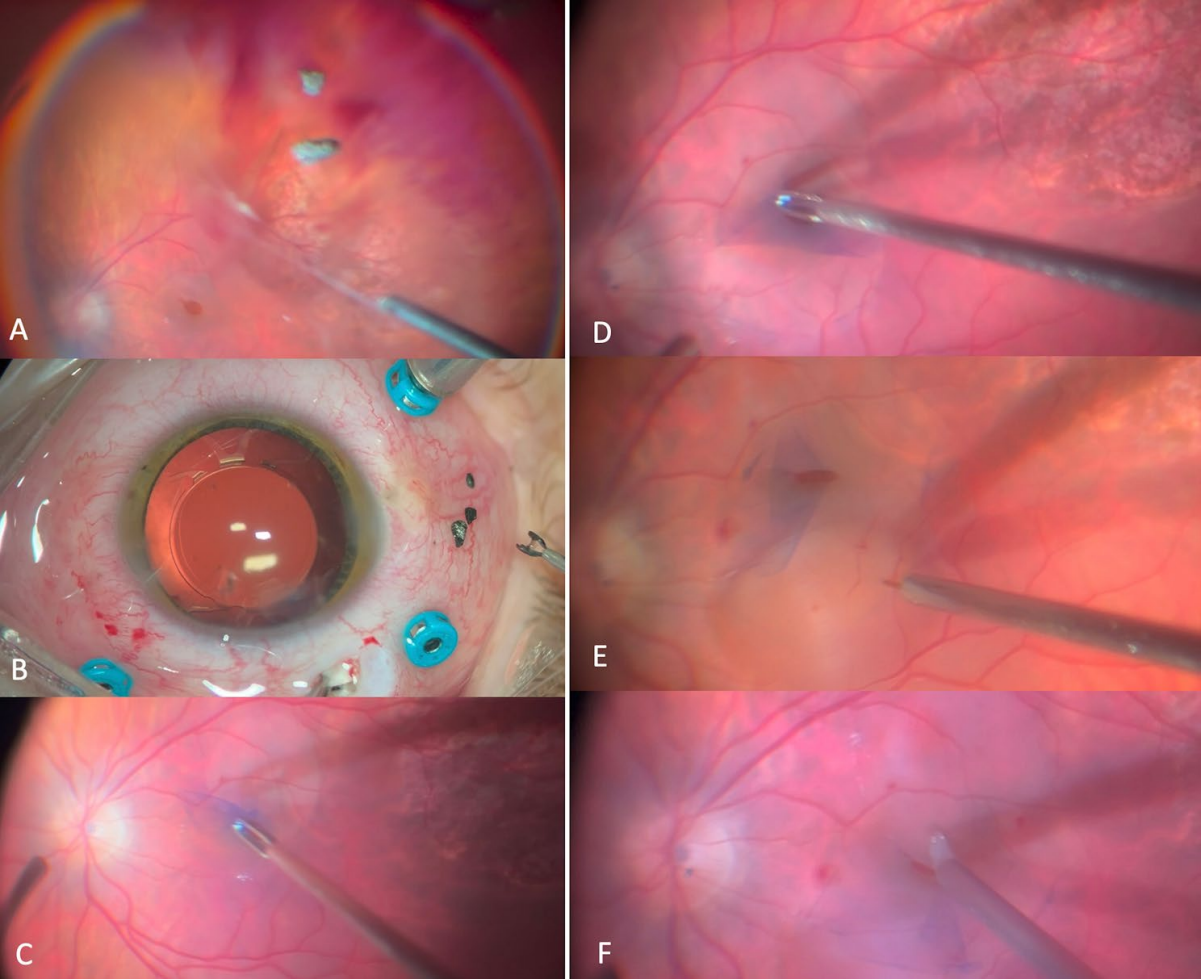
Case 1. Steps of the surgery: (**A**). Vitreous and posterior hyaloid removal; (**B**). IOFBs removal through pars plana incision; (**C**). Inferior ILM peeling; (**D**). Superior ILM inverted flap; (**E**). Subretinal fluid application with 41G cannula; (**F**). Centripetal retinal displacement with Tano diamond dusted membrane scraper

Four eyes (28.6%) with MH were secondary to an open-globe, whereas ten eyes (71.4) were related to a closed-globe injury. Blast trauma was the cause of injury in all (100%) BRTMH cases. In one eye, MH was concurrent with presence of three intravitreal foreign bodies (Figs. 1 and 2, Supplemental Digital Content 1). None of the patients included in the case series were wearing eye protection.

The median (IQR) OTS was 68 (56–75), while the time interval from injury to the surgery was 41 (19 to 71) days. In 13 of 14 patients, the holes were primary, while one patient had two holes: one persistent hole after a failed surgery at the local hospital, and the second eccentric one as a result of iatrogenic injury. Table 1 summarizes the demographics of the patient population and the details of the circumstances causing BRTMH.

**Table 1:** Preoperative characteristics of the patients with combat Blast-Related traumatic Full-Thickness macular holes

Variables	Value
Age, years, median (IQR)	32 (25.5–43.0)
Operated eye, n/N (*P* ± 95CI%):	
Right Left	7/14 (50 ± 27.2) 7/14 (50 ± 27.2)
Type of Globe injury, n/N (*P* ± 95CI%):	
Open-globe Closed-globe	4/14 (28.6 ± 24.6) 10/14 (71.4 ± 24.6)
Association of ocular trauma with ocular adnexal and orbital injuries, n/N (*P* ± 95% CI)	9/14 (64.3 ± 26.0)
Association of ocular trauma with other head, neck, face or systemic injuries, n/N (*P* ± 95% CI)	11/14 (78.6 ± 22.3)
Retinal Detachment, n/N (*P* ± 95% CI)	3/14 (21.4 ± 22.3)
The median time from the moment of injury, days, median (IQR)	41 (19–71)
Binocular Macular Hole, n/N (*P* ± 95% CI)	1/13 (7.6 ± 14.0)
Ocular Trauma Score, median (IQR)	68 (56–75)
Preoperative BCVA decimal, median (IQR)	0.07 (0.02–0.10)
Preoperative BCVA logMAR, median (IQR)	1.2 (1.0–1.8)
Minimum Linear Diameter (µm), median (IQR): < 400 μm, n/N (*P* ± 95CI%) 400–600 μm, n/N (*P* ± 95CI%) > 600 μm, n/N (*P* ± 95CI%)	682 (532–889) 2/14 (14.3 ± 19.0) 3/14 (21.4 ± 22.3) 9/14 (64.3 ± 26.0)

Abbreviation: N - sample size; n - number of patients; (*P* ± 95CI%) - frequency and 95% confidence interval; IQR - interquartile range (25–75%)

A combined procedure (phacoemulsification and intraocular lens implantation in combination with 25G PPV) was performed in 7 eyes (50%) where lens opacity made it difficult to examine the fundus. MH closure was achieved in all cases (100%) after the primary surgery.

**Fig. 3:**
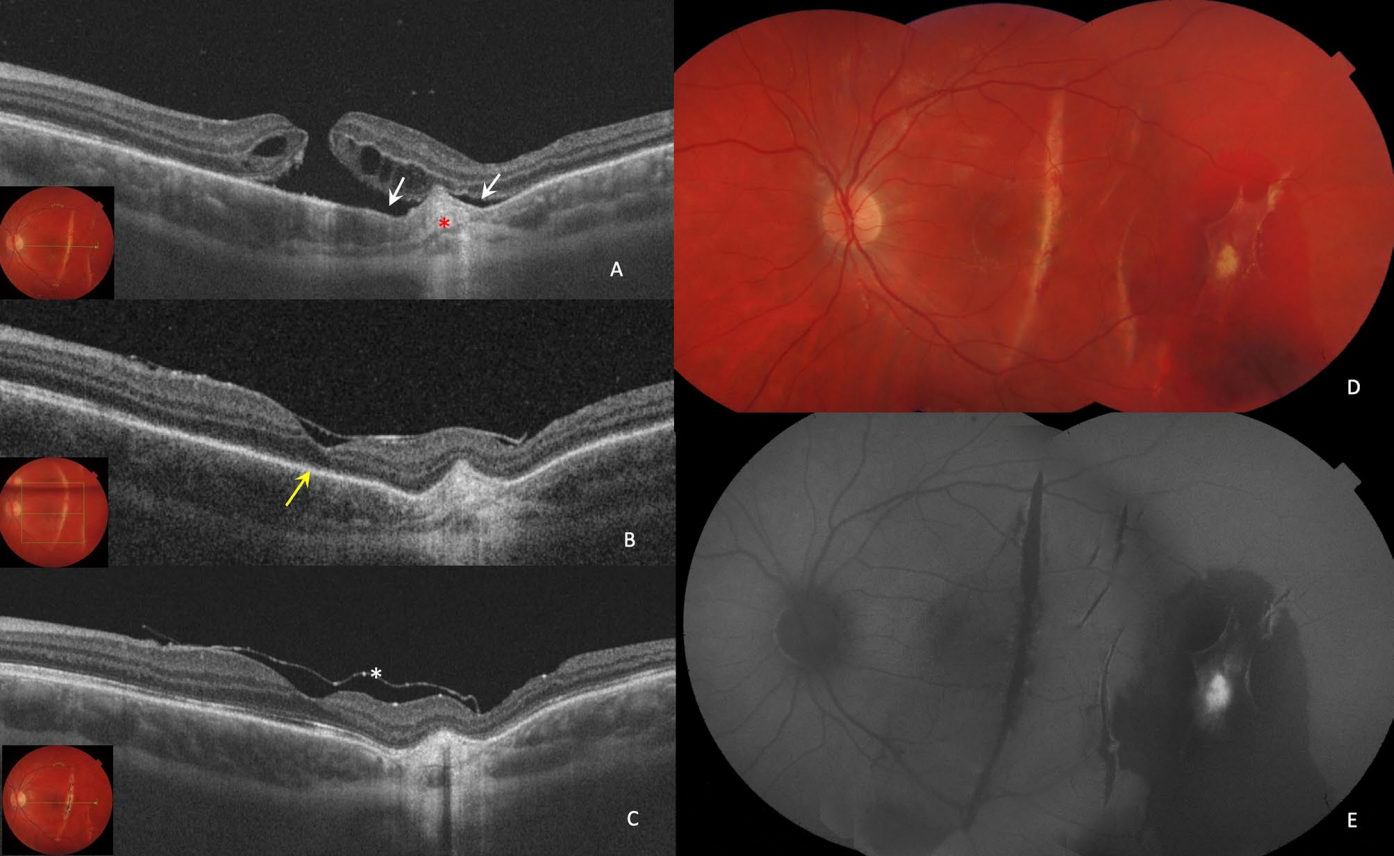
Case 2: 20-year-old man with BRTMH was treated with vitrectomy 25G and inferior ILM peeling + superior ILM inverted flap + subretinal fluid application + centripetal retinal displacement and SF_6_ gas. (**A**). His preoperative MLD was 253 μm, OCT demonstrates local temporal neuroepithelium detachment (white arrows) and subretinal fibrosis as the result of choroidal rupture (red asterisk). Preoperative BCVA was 0.1 decimal (**B**). Seven days after surgery, the MH was closed, the ELM line restored but the EZ line was noticeably interrupted (yellow arrow) (**C**). One month after surgery, the ELM and EZ lines completely restored. ILM flap above the retina (white asterisk). The BCVA was 0.6 decimal. (**D**, **E**). Mosaic fundus photo and FAF images at 1 Month postoperative

**Fig. 4:**
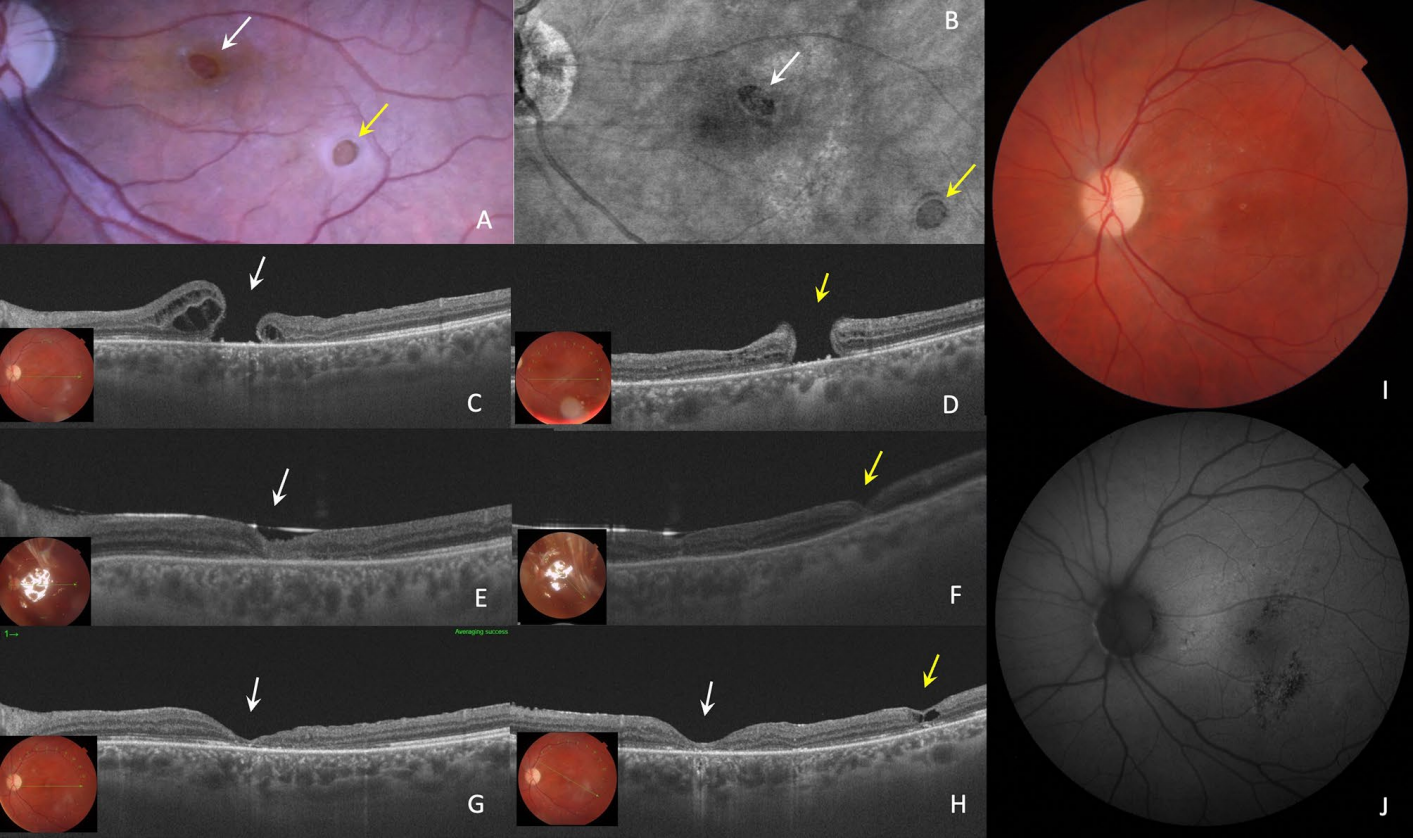
Case 3: (**A**). Fundus photo and (**B**). enface OCT image of 45-year-old man with persistent BRTMH (white arrow) previously operated with conventional PPV and ILM peeling (in another clinic) and one more iatrogenic eccentric macular hole (yellow arrow). (**C**, **D**). His preoperative MLD of two holes were 661 μm and 503 μm respectively. Preoperative BCVA was 0.04 (decimal). 25G PPV was performed with hydrodissection technique and centripetal retinal displacement for recurrent macular hole. For eccentric iatrogenic hole temporal inverted ILM flap was performed. (**E**, **F**). OCT 1 day after surgery: both MHs were closed, therefore a face down position was stopped. (**G**). One month after surgery, the BCVA was 0.1 (decimal), OCT reveled that the ELM line was restored but the EZ line was disrupted. (**H**). OCT at 1 M postop: In closed eccentric macular hole cystic space in inner retina was detected. (**I**). Fundus photo demonstrates signs of optical neuropathy: pale optic disk, (**J**). 1 Month postop: on FAF image hypofluorescence foci in fovea and macula

Circular ILM peeling (CIP) with fluid drainage through the hole (FDH) was used in 4 cases (28.5%), while inferior ILM peeling (IIP) with superior inverted ILM flap (SIIF) in combination with subretinal fluid application (SFA), centripetal retinal displacement (CRD) with or without dissection of the subretinal adhesion (DSA) was performed in six cases (42.8%) (Fig. 3, Supplemental Digital Content 2 and 6). Multilayer inverted ILM flaps (MIIF) combined with SFA, CRD and DSA was performed in one case (7.1%). For recurrent MH, perifoveal hydrodissection and CRD techniques were used, and for eccentric hole temporal inverted ILM flap was performed (Fig. 4, Supplemental Digital Content 3).

**Fig. 5:**
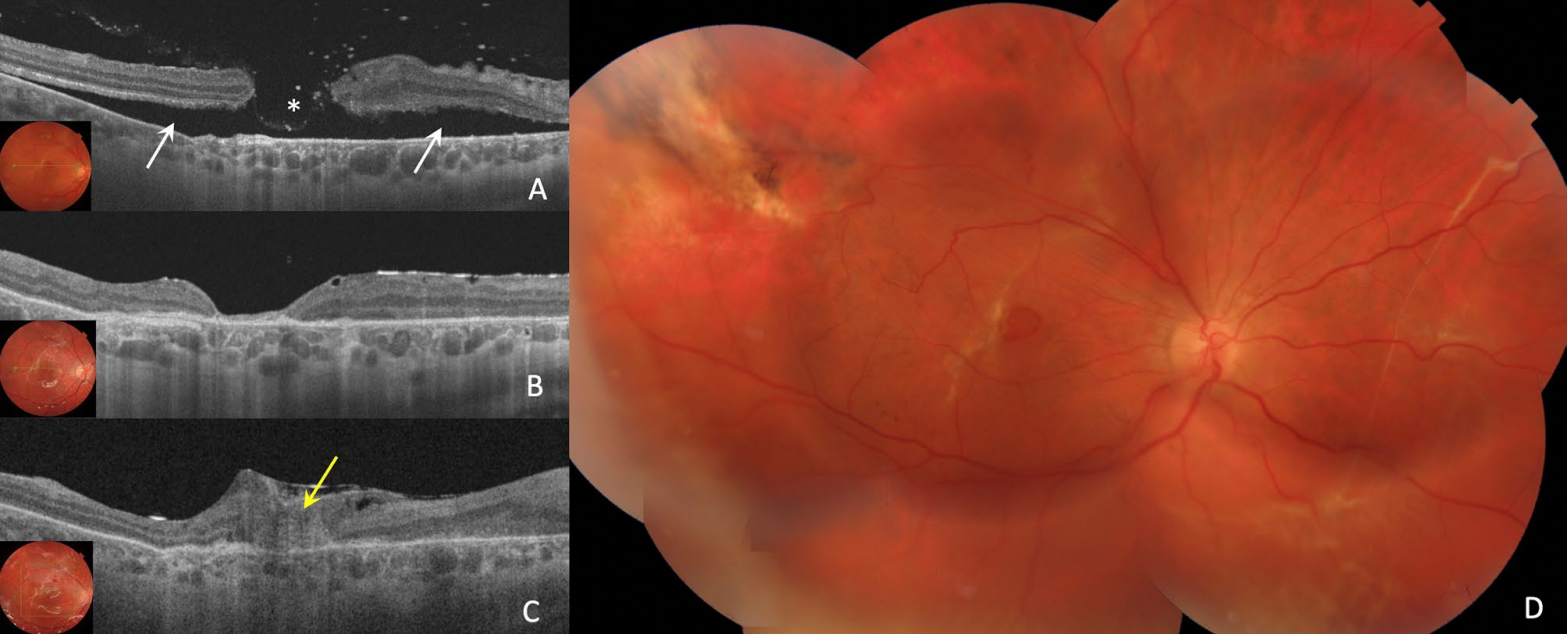
Case 4: 43-year-old man with BRTMH associated with retinal detachment was treated with phacovitrectomy 25G and multilayer inverted ILM flap + centripetal retinal displacement and silicon oil tamponade. (**A**). His preoperative MLD was 1007 μm, OCT demonstrates neuroepithelium detachment (white arrows) and vitreous incarceration inside the hole (white asterisk). Preoperative BCVA was 0.1 decimal (**B**). At 1 Month after surgery, the MH was closed as thin layer of glial tissue (modified ILM membrane) without detectable ELM and EZ lines. (**C**). Three months after primary surgery and two weeks after silicon oil extraction, OCT revealed massive glial proliferation at the place of ILM flap with outer retinal layers degeneration (yellow arrow). The BCVA was 0.3 decimal. (**D**). Preoperative mosaic fundus photo demonstrates vitreous hemorrhages, supertemporal peripheral choroidal rupture and subretinal fibrosis

**Fig. 6:**
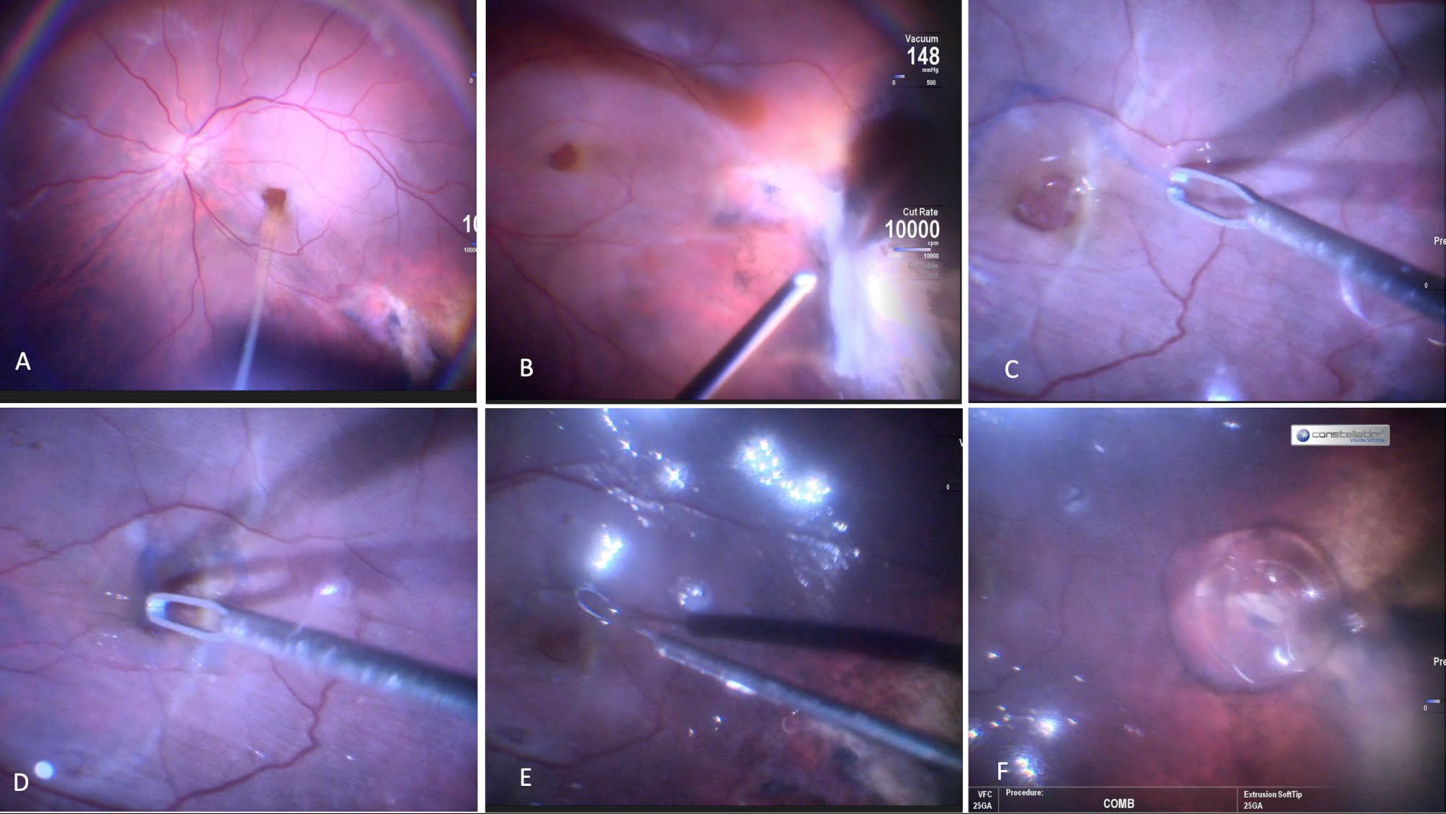
Case 4: Steps of the surgery: (**A**). Core vitrectomy with drainage of subretinal fluid through macular hole; (**B**). Vitreous base shaving; (**C**,**D**). Multilayer ILM flap preparing; (**E**). Centripetal retinal displacement and ILM flaps reposition with Flex loop (Alcon); (**F**). Silicon oil injection

In cases where MHs were accompanied by retinal detachment, a combination of MIIF with CRD was used in one case (7.1%); IIP with SIIF and CRD was used in two cases (14.2%) (Figs. 5 and 6, Supplemental Digital Content 4). For intraocular endotamponade, a gas–air mixture of sulfur hexafluoride (SF_6_ 20%) in seven cases (50%), 16% C_2_F_6_ in four cases (28.5%) and silicon oil (SO) 5000 cSt in three cases (21.4%) were used. The intraoperative characteristic of each case is listed in Table 2.

**Table 2:** Intraoperative characteristics patients with combat blast-related traumatic FTMH

Cases	Surgery	Macular hole manipulations	Tamponade	Macular Findings	Nonmacular Findings	Hole closure
Case 1	PPV + IOL	MIIF + CRD	SO	RRD, VH, RPEA	VH, SC, RRD, SF	Yes
Case 2	PPV	IIP + SIIF + SFA + CRD	SF_6_	SF	SRH, SF	Yes
Case 3	PPV	IIP + SIIF + CRD	SO	RRD, VH, SRH, SF, RPEA	VH, RRD, SC	Yes
Case 4	PPV	CIP + FDH	SF_6_	N/A	SRH, SF	Yes
Case 5	PPV + IOL	IIP + SIIF + SFA + CRD	C_2_F_6_	N/A	IOFBs, IOFBIS, SC	Yes
Case 6	PPV	SFA + CRD (for persisting hole) TIIF (for eccentric iatrogenic hole)	SF_6_	RPEP	TON, RPEA	Yes
Case 7	PPV + IOL	IIP + SIIF + SFA + DSA + CRD	SF_6_	RPEP	N/A	Yes
Case 8	PPV + IOL	MIIF + SFA + DSA + CRD	C_2_F_6_	RPEP	SC, RPEP	Yes
Case 9	PPV	IIP + SIIF	SF_6_	RPEA, SC	VH, SRH, SC	Yes
Case 10	PPV	CIP + FDH	SF_6_	VH, RPEP	TC, TM, VH, SRH, SC	Yes
Case 11	PPV	CIP + FDH	SF_6_	VH, RPEP	TC, TM, VH, SRH, CS	Yes
Case 12	PPV + IOL	IIP + SIIF + SFA + DSA + CRD	SO	RPEP	SC	Yes
Case 13	PPV + IOL	CIP + FDH	C_2_F_6_	RPEP	SC, SRH	Yes
Case 14	PPV + IOL	IIP + SIIF + CRD	C_2_F_6_	RRD, VH, RPEP	RRD, VH, SC, CH	Yes

Abbreviations: MIIF Multilayer Inverted ILM Flaps; CIP Circular ILM Peeling; IIP Inferior ILM Peeling; SIIF Superior Inverted ILM Flap; TIIF Temporal Inverted ILM Flap; FDH Fluid Drainage through the Hole; CRD Centripetal Retinal Displacement; SFA Subretinal Fluid Application; DSA Dissection of Subretinal Adhesion; SO Silicon oil

IOFB Intraocular foreign body; IOFBIS Intraocular foreign body impact site; CS Corneal scar; TC Traumatic Cataract; TM Traumatic mydriasis; RRD Rhegmatogenous retinal detachment; SC Sclopeteria; RPEA Retinal pigment epithelium atrophy; RPEP Retinal pigment epitheliopathy; SRH Subretinal hemorrhage; SF Subretinal fibrosis; VH Vitreous hemorrhage; CH Choroidal hemorrhage; TON Traumatic optic neuropathy; N/A Nonapplicable

Mean BCVA significantly improved from median (IQR) 0.07 (0.02–0.10) decimal to 0.3 (0.14–0.40) decimal at one month, and from 1.2 (1.0–1.8) logMAR, median (IQR) to 0.5 (0.4–0.9) logMAR (Table 2).

**Table 3:** Anatomical and functional results in patients with combat Blast-Related traumatic Full-Thickness macular holes after surgery

Variables	Value
Macular hole closed after PPV, n/N (*P* ± 95% CI)	14/14 (100 ± 21.5)
Phacoemulsification + IOL in combination with PPV, n/N (*P* ± 95% CI)	7/14 (50.0 ± 27.2)
Tamponade, n/N (*P* ± 95% CI):	
Silicon oil Gas	3/14 (21.4 ± 22.3) 11/14 (78.6 ± 22.3)
BCVA (decimal), at 1 month, median (IQR)	0.3 (0.14–0.40) *
BCVA logMAR at 1 month, median (IQR)	0.5 (0.4–0.9) *
Follow-up duration, days, median (IQR)	75 (43–175)
Intraoperative complications, n/N (*P* ± 95CI%): Transitory arterial occlusion Retinal tear Preretinal haemorrhage	3/14 (21.4 ± 22.3) 1/14 (7.1 ± 14.0) 1/14 (7.1 ± 14.0) 1/14 (7.1 ± 14.0)
Postoperative complications, n/N (*P* ± 95CI%): Retinal detachment ILM flap proliferation Vitreous hemorrhage IOL subluxation Persistent IOP elevation Endophthalmitis	1/14 (7.1 ± 14.0) 0/14 (0 ± 21.5) 1/14 (7.1 ± 14.0) 0/14 (0 ± 21.5) 0/14 (0 ± 21.5) 0/14 (0 ± 21.5) 0/14 (0 ± 21.5)

Note: * - visual acuity after surgery is statistically significantly (*p* = 0.001) different from preoperative values, Wilcoxon signed-rank test W = 3,3

Abbreviation: N - sample size; n - quantity of patients; (*P* ± 95CI%) - frequency and 95% confidence interval; IQR - interquartile range (25–75%)

Time from the moment of injury (days) showed no statistically significant correlation with the visual acuity at one month after surgery (ρ = − 0.43, *p* = 0.07), while there was a direct correlation of the postoperative visual acuity at 1 month with the OTS score (ρ = 0.51, *p* = 0.03), preoperative visual acuity (LogMar) (ρ = 0.72, *p* = 0.002), and an inverse correlation with the size of the MH (ρ = -0.63 *p* = 0.008).

The factors that showed statistically significant influence on the postoperative (at 1 month) BCVA being ≥ 0.3 were: preoperative BCVA more than 0.1 decimal (*p* = 0.03), associated OTS ≥ 70 (*p* = 0.02) and MLD of the MH < 400 μm (*p* = 0.03) (Table 3).

**Table 4:** Factors affecting the achievement of postoperative best corrected visual Acuity ≥ 0.3

Risk Factors	Visual Acuity ≥ 0.3		р-value
	Yes	No	
Days Before Surgery ≤31, yes/no	5/3	1/5	0.12
Closed-globe injury, yes/no	5/3	5/1	0.41
Ocular Trauma Score ≤ 70, yes/no	1/7	5/1	0.02*
Sclopetaria, yes/no	5/3	4/2	0.66
OcularAdnexal and Orbital Injuries, yes/no	5/3	4/2	0.66
Head, Neck, Face or Systemic Injuries,	6/2	5/1	0.62
Preoperative BCVA ≤ 1.0 logMAR yes/no	3/5	6/0	0.03*
Minimum Diameter Macular Holes <400, yes/no **	0/9	3/2	0.03*
Tamponade Gas, yes/no	7/1	4/2	0.39
Retinal Detachment, yes/no	1/7	2/4	0.39

Note: * - Fisher’s Exact Test was used, one-tailed association. ** - For Postoperative Visual Acuity greater than 0.3

Factors such as closed globe injury, time from the moment of injury to surgery, type of tamponade, presence of retinal detachment, association of ocular trauma with adnexal and orbital injuries and concomitant head, neck, face or systemic injuries did not show a statistically significant impact on achieving BCVA ≥ 0.3 at one month postoperatively.

Twelve of fourteen eyes (85.7%) with BRTMHs had other posterior globe injuries: vitreous haemorrhage (five eyes, 35.7%), retained intraocular foreign bodies (one eye, 7.1%), rhegmatogenous retinal detachment (three eyes, 21.4%), traumatic chorioretinitis sclopetaria (nine eyes, 64.2%), retinal pigment epitheliopathy (eleven eyes, 78.5%), traumatic optic neuropathy (one eye, 7.1%), subretinal fibrosis (three eyes, 21.4%), subretinal hemorrhage (six eyes, 42.8%).

Intraoperative complications included one case (7.1%) of transitory arterial occlusion (Supplemental Digital Content 5), one case (7.1%) peripheral retinal tear (Supplemental Digital Content 1) and one case (7.1%) preretinal haemorrhage. We did not observe any serious postoperative complications except in one case of macular pucker development at the ILM flap site (Fig. 5).

## Discussion

This study reports characteristics and outcomes of surgery for combat-related traumatic FTMH at a single civil quaternary reference center responsible for treating the majority of such injuries.

The variety and severity of injury patterns due to using heavy weapon systems and logistical features of the Ukrainian military medical system, which meets the standards of NATO, enables us to correctly compare our experiences to previously published retrospective studies dealing with military conflicts in Iraq and Afghanistan and well-established practice patterns.

In our study sixteen of the 365 eyes (4.4%) were diagnosed with a traumatic FTMH. From the fourteen eyes included, ten eyes (71.4%) with a BRTMH were secondary to a closed-globe, whereas four eyes (21.6%) were related to an open-globe injury that coincide with data in the literature on the leading role of contusions as the cause of the traumatic FTMH [5, 7, 12].”

According to our data, sixteen of the 365 eyes (4.4%) were diagnosed with a traumatic FTMH. From the fourteen eyes included, ten eyes (71.4%) with a BRTMH were secondary to a closed-globe, whereas four eyes (21.6%) were related to an open-globe injury that coincide with data in the literature on the leading role of contusions as the cause of the traumatic FTMH [12].

We also confirm that combat eye trauma almost always is a complex eye polytrauma where the eye is only one of the components of systemic polytrauma. Our series demonstrates that the association of ocular trauma with ocular adnexal and orbital injuries was in 64.3% cases, and association of ocular trauma with head, neck, face or other systemic injuries was in 78.6% cases. The data we received confirm the results by the Zhupan B. et al. study, where the authors demonstrated high rate (81.4%) of multiple combat-related eye injuries in the Ukraine-Russia War likely due to wide use of high-kinetic-energy weapons with high-velocity fragments from explosive munitions, which systems have wide-area effects [3].

Combat eye injury after blast trauma due to a massive amount of kinetic energy resulted in a spectrum of concomitant macular and non-macular damage that determines the outcome. Due to the individual uncertainty of the force and direction imposed on the eye, and the individual structural features of the eye, exact location, extent of the retinal injury and rate of progression of BRTMH are still difficult to predict clinically.

In our series, all patients (100%) had other posterior segment injuries. There was a high range of macular findings from retinal pigment epitheliopathy to subretinal fibrosis, subretinal hemorrhages or RPE atrophy. The patients in this study also had associated traumatic retinal detachment, optic neuropathy, sclopetaria, and choroidal hemorrhage.

Our data coincide with the results of a previous study where the authors reported traumatic MHs with other posterior segment globe injuries [5, 7].

The variety and severity of eye injuries due to combat trauma are the result of both the multifactorial action of the blast wave front, and the lack of eye protection. It is generally known that Military Combat Eye Protection (MCEP) decreases both the incidence and severity of eye injuries [13, 14]. During the Iraq war (2004–2005), less than 10% of combatants treated for eye injuries admitted to wearing eye protection device at the time of injury [15]. One of the reasons for low compliance has been the restricted field of vision and fogging from eye protection. In our series, none of the patients was wearing goggles at the time of the injury, and this is an extremely important issue. This was also the main reason why our wounded patients had a high level of penetrating injuries, IOFBs and binocular lesions (in press).

The effectiveness of modern PPV in the treatment of idiopathic MHs exceeds 90%, but traumatic MH has long been known to have the worst prognosis, regardless of the surgical technique [16]. Miller et al. [17] published data regarding the use of PPV for traumatic MH with an overall single-operation success rate of closure in 83% of cases. Various surgical approaches and adjuncts have been used in surgery for traumatic MH to gain the reposition of the hole margin, including radial retinal incisions at the rim of the hole, and the use of plasmin, transforming growth factor–b2, platelets, and autologous serum [18–20].

In relation to the anatomical and functional results, BRTMHs are the most difficult category to treat due to the severe concomitant damage to the retina, RPE, choroid and optic nerve. Until now, there has been no standard surgical approach to treat traumatic MH related to combat trauma mainly due to the small number of published series and cases.

Weichel ED et al.^7^. demonstrated a closure rate of 67% (8 of 12) in combat traumatic MH surgery. All eyes in their series underwent a standard three-port 20- or 25-gauge PPV with creation of a posterior vitreous detachment, but the ILM was not removed.

In a series by Phillips BN et al.^5^, five eyes with combat related traumatic MH underwent surgical repair, but authors could not indicate the anatomical outcome of the surgery, possibly because OCT examination was not performed in two cases. The surgical technique included vitrectomy with membrane peeling and gas tamponade (three cases) and silicone oil tamponade (one case with retinal detachment) without specifying whether the ILM was removed.

Although, to the best of our knowledge, we present the largest series of eyes operated with combat-related traumatic MHs and 100% closure rate after primary surgery, the small number of examined eyes and the short postoperative follow-up period do not allow us to draw definitive conclusions regarding the anatomical and functional outcomes of treatment.

A preoperative evaluation and identification of reliable OCT-based MH biomarkers is crucial for predicting surgical outcomes and choosing the optimal approach in BRTMH surgery. The values ​​of the MLD, Basal Diameter like the presence and severity of epi-/sub-retinal proliferation can determined the choice of the surgical approach accordingly.

We strongly believe that MH closure by “primary intention” should be considered the optimal and desired result, which gives favorable anatomical and functional outcomes. This is possible only with an initial minimal tissue defect, maximum approximation of the wound edges and the absence of any adjuvants in the wound [21].

Therefore, for holes less than 500 μm with no signs of subretinal adhesion, our main surgical approach was: circular ILM peeling with fluid drainage through the hole. For more difficult cases (MLD > 500 μm, signs of strong subretinal adhesion) subretinal fluid application with centripetal retinal displacement and mechanical dissection of subretinal adhesion if needed were performed. This surgical step was very important to achieve a sufficient foveal detachment with further mobilization of the MH edges [22]. Perifoveal centripetal macular displacement of the detached retinal tissue was performed as a gentle massage using a backflush cannula.

In the particularly difficult cases (MLD > 800 μm with signs of diffuse subretinal adhesion), when it was assessed impossible to achieve the necessary displacement of the foveolar tissue, and there was no hope for hole healing by primary intension, we used various types of inverted flap techniques (superior, temporal or multilayers) described earlier by Michalewska Z. et al. [23]. In such cases, the holes were closed by “secondary intention” with a later and abnormal restoration of the outer layers of the retina (ELM, EZ) which reduced the functional outcome.

The preferred type of tamponade in the present series was gas (20% SF_6_ or 16% C_2_F_6_), while silicon oil was used only in two cases of concomitant retinal detachment and “giant” MHs (MLD 1132 μm).

The timeframe of postoperative positioning after MH surgery remains controversial and strong evidence is still not available for the optimal duration of the prone positioning to achieve MH closure [24, 25]. Nevertheless, prone positioning is considered uncomfortable for the patients and may lead in some cases to pressure sores or neuropathy [26, 27]. While the ability to maintain a prolonged face-down positioning should be limited and undesirable for the wounded in most cases, we recommended it for only 24 h.

We strongly suggest that combining several independent surgical techniques may synergistically enhance their effectiveness while minimizing individual limitations and reducing failure rates [28]. In the current study, such approaches allow us to avoid unwanted reoperations even for MHs with median MLD 682 μm.

The functional outcome in our study at 1 month (median decimal BCVA was 0.3 and 57% eyes achieved BCVA ≥ 0.3) was better than in the Weichel et al. study [7], where only 33.3% of the eyes achieved BCVA ≥ 0.3 (decimal equivalent), or in the study by Phillips et al.^5^, where only three of five eyes showed an improvement in visual acuity after surgery, and the ffinal visual acuity was 20/80 and 20/150 for the 2 MHs being observed. Our data support the opinion of other studies that MLD < 400 μm statistically significantly influenced post-operative vision.

The better visual outcomes observed in our study may be attributed to a higher proportion of eyes with successful MH closure and better preoperative visual acuity, both of which were found to have a statistically significant influence on post-operative vision.

Although BCVA of all eyes in this study was improved after surgery with a significant pre-operative-to-post-operative difference at one-month, the visual outcome was highly variable despite MH closure in the majority of our patients undergone concomitant globe injuries.

Our data does not confirm a statistically significant correlation of the post-operative BCVA ≥ 0.3 at 1 month with factors such as: closed globe injury, time from the moment of injury to surgery, type of tamponade, presence of retinal detachment, association of ocular trauma with adnexal and orbital injuries and concomitant head, neck, face or systemic injuries. However, the small sample size of this study does not allow us to draw a definitive conclusion. Further multicentred studies are needed to clarify these issues.

OTS proposed by Kuhn et al., is one of the most commonly used systems for estimating the probability of vision recovery following closed-globe and open-globe injuries. Although there is consensus on the prognostic predictive value of the OTS in general, there is variation on this value for specific OTS categories [10]. The previous data suggest that the OTS can also be used to predict visual outcomes in both combat-related and non-combat-related globe injuries and traumatic MHs [29–31]. The current study demonstrates that the OTS can be used to predict favorable visual outcomes in combat-related traumatic MHs.

Regarding the intraoperative complications in our series, we observed single cases of transient branch central retinal artery occlusion, peripheral retinal tear and pre-retinal hemorrhage which have not led to adverse consequences. There were no cases with post-operative retinal detachment, persistent IOP elevation, or endophthalmitis. Among post-operative complications, we observed one case of ILM flap proliferation after applying the multilayer ILM inverted flap technique, which led to decreased vision. Such complication has been reported previously by Kanda et al., as “macular pucker” in two patients after using the inverted ILM flap technique [32].

This study has several limitations. First, it is retrospective in nature with a median follow-up period of 75 days, which may lead to an underestimation of the true functional outcomes. The lack of long-term follow-up for some wounded individuals limits the ability to evaluate the restoration of the external retinal layers (ELM/EZ) and observe the progression of vision-affecting complications, particularly considering that blast-related ocular trauma may evolve over time.

The relatively small sample size (13 cases, 14 eyes) also limits the statistical analysis of the findings. Furthermore, all surgeries were performed at a single center by one experienced vitreoretinal surgeon, which may affect the external validity and reproducibility of the results. The specific context of war-related injuries and a predominantly military patient population further restricts the applicability of the findings to civilian settings. In addition, the absence of a control group makes it difficult to directly compare the effectiveness of the surgical techniques used. Post-operative monitoring was only possible during the period prior to patients’ return to duty or transfer to the Veterans Affairs medical system, resulting in variations in the follow-up length and data completeness. Finally, the individualized application of several surgical techniques introduces heterogeneity, making it challenging to attribute outcomes to any single component of the surgical strategy. Conducting clinical research under wartime conditions poses significant challenges, making it difficult to maintain ideal study protocols and follow-up consistency.

## Conclusion

Blast-related traumatic MHs resulting from combat-related eye injuries are almost invariably accompanied by severe globe damage and additional non-ocular trauma. Once the patient is stabilized, surgical repair of BRTMH has proven to be a highly effective treatment option. Although there is currently no consensus on the optimal surgical technique, a synergistic combination of approaches—particularly those that minimize the need for post-operative face-down positioning—can lead to excellent anatomical outcomes while remaining safe and feasible for the wounded patients. Nevertheless, the functional effectiveness of these combined techniques warrants further investigation.

## Supplementary Information

Below is the link to the electronic supplementary material.

Supplementary Material 1

Supplementary Material 2

Supplementary Material 3

Supplementary Material 4

Supplementary Material 5

Supplementary Material 6

## Data Availability

No datasets were generated or analysed during the current study.

## Abbreviations

FPV-drones: First-person view drones
IOFBs: Intraocular foreign bodies
MH: Macular hole
BCVA: Best corrected visual acuity
BRTMH: Blast-related traumatic macular hole
FTMH: Full-thickness macular hole
OTS: Ocular Trauma Score
BETT: Birmingham Eye Trauma Terminology
RAPD: Relatively afferent pappillary defect
NATO: North Atlantic Treaty Organization
THBs: Territorial hospital bases
MMCCs: Military Medical Clinical Centers
SS-OCT: Swept source optical coherence tomography system
MLD: Minimal Linear Dimension
RPE: Pigmented epithelium
ILM: Internal limiting membrane
PPV: Pars plana vitrectomy
DD: Disk diameters
BSS: Balanced salt solution
SF6: Sulfur hexafluoride
C2F6: Hexafluoroethane
FDP: Face down position
PFCL: Perfluorocarbon liquid
SO: Silicon oil
NSAID: Non-steroidal anti-inflammatory drugs
logMAR: Logarithm of minimal angle of resolution
CIP: Circular ILM peeling
FDH: Fluid drainage through the hole
IIP: Inferior ILM peeling
SIIF: Superior inverted ILM flap
SFA: Subretinal fluid application
CRD: Centripetal retinal displacement
DSA: Dissection of the subretinal adhesion
MIIF: Multilayer inverted ILM flaps
MCEP: Military Combat Eye Protection
ELM: External limiting membrane
EZ: Ellipsoid zine
